# Rekonstruktion von metastasenbedingten Defekten des Azetabulums mittels der MRS-C-Abstützschale

**DOI:** 10.1007/s00064-023-00834-6

**Published:** 2023-11-03

**Authors:** S. Koob, H. Kohlhof, T. M. Randau, D. C. Wirtz

**Affiliations:** 1 Klinik und Poliklinik für Orthopädie und Unfallchirurgie, Universitätsklinikum Bonn, Venusberg-Campus 1, 53127 Bonn, Deutschland; 2 Unfall‑, Hand- und Orthopädische Chirurgie, St. Antonius Krankenhaus Köln, Köln, Deutschland

**Keywords:** Knochenmetastasen, Pathologische Fraktur, Hüftfraktur, Verbundosteosynthese, Palliative Chirurgie, Metastatic bone disease, Pathologic fracture, Hip fracture, Compound osteosynthesis, Palliative surgery

## Abstract

**Operationsziel:**

Stabilisierung des metastatisch befallenen Azetabulums mit einer modularen, zementaugmentierten Abstützschale zur Remobilisation von onkologischen Patienten auch im fortgeschrittenen Krankheitsstadium.

**Indikationen:**

Metastasenbedingte azetabuläre Defektsituationen (Metastatic Acetabular Classification, MAC 2–4) bei mittel- und langfristiger Überlebensprognose des Patienten.

**Kontraindikationen:**

Starke Einschränkung der Überlebensprognose (< 6 Wochen), persistierendes lokales Infektgeschehen, Vorliegen eines primären Knochentumors mit kurativem Therapievorgehen, ausgeprägte Beckendiskontinuität, laufende wundheilungskompromittierende Chemo- oder Immuntherapie.

**Operationstechnik:**

Standardhüftgelenkzugang. Resektion bzw. Kürettage der Azetabulummetastase und vorsichtiges Auffräsen zur Einbringung der Probeschale. Nach Festlegung der Schalengröße, Vorbohren der Dom- und Laschenschrauben und Einbringen des Zementes durch die zentrale Schalenöffnung in den azetabulären Defekt. Anschließend Festdrehen der Schrauben im Sinne einer Verbundosteosynthese. Einbringen einer modularen Pfannenkomponente oder Einzementierung eines tripolaren Pfannensystems in Verbindung mit einer Standardfemurschaftprothese oder einem proximalen modularen Femurersatz.

**Weiterbehandlung:**

Faden‑/Klammerentfernung nach 10 Tagen. Mobilisation unter schmerzadaptierter Vollbelastung. Gangschulung an 2 Unterarmgehstützen. Physiotherapie zur Kräftigung der hüftumgreifenden Muskulatur. Je nach Tumorboard-Beschluss ggf. adjuvante Radiatio nach Abschluss der primären Wundheilung und Fortsetzung der systemischen Therapie.

**Ergebnisse:**

Im Zeitraum 2012 bis 2019 wurden 14 Patienten mit einem metastasenbedingten azetabulären Defekt mit einer zementaugmentierten Abstützschale („MRS-TITAN® Comfort“, MRS-C, Peter Brehm GmbH, Weisendorf, Deutschland) versorgt. Der Harris-Hip-Score verbesserte sich durchschnittlich um 23,2 Punkte bei einem mittleren Überleben der Patienten von 9,7 Monaten aufgrund der reduzierten Patientenprognose bei Karzinomerkrankung. Bei 13 der 14 implantierten Abstützschalen waren keine Folge- oder Revisionseingriffe notwendig. In einem Fall kam es zu einem weichteildefektbedingten periprothetischen Infekt und zur Explantation der Abstützschale.

## Abstract (en)

**Objective:**

Stabilization of metastatic acetabular defects with a bone cement-augmented revision support cup for remobilization of oncological patients in advanced cancer stages.

**Indications:**

Metastatic acetabular defects (Metastatic Acetabular Classification, MAC 2–4) in patients with a prognostic medium or long-term survival.

**Contraindications:**

Highly limited survival due to metastatic disease (< 6 weeks). Local bone or soft tissue infection. Primary bone tumor with curative treatment option. Advanced pelvic discontinuity. Recent wound compromising systemic therapy.

**Surgical technique:**

Standard hip approach. Curettage of the metastatic defect and careful reaming of the acetabulum before insertion of the cup. Predrilling of the dome und flange screws before application of the bone cement through the center hole of the implant and filling of the acetabular defect. Complete insertion of the screws for compound osteosynthesis. Implant of a modular inlay or dual mobility system.

**Postoperative management:**

Full weight bearing or mobilization with two crutches according to the level of pain. Adjuvant local radiation therapy after wound consolidation. Continuation of systemic therapy according to tumor board decision.

**Results:**

Between 2012 and 2019, we treated 14 patients with metastatic acetabular defects using the modular revision support cup „MRS-TITAN® Comfort“, MRS-C, Peter Brehm GmbH, Weisendorf, Germany) at our institution. Mean Harris Hip Score improvement was 23.2 with a mean patient’s survival of 9.7 months due to the reduced cancer-related prognosis; 13 of the 14 implants endured the patient’s prognosis. One implant had to be removed due soft tissue defect-related periprosthetic joint infection.

## Vorbemerkungen

Das Becken und Azetabulum sind nach der Wirbelsäule die häufigsten Lokalisationen von stabilitätsgefährdenden Karzinommetastasen und stellen den tumororthopädischen Behandler in der Vielzahl der Fälle vor eine operative Herausforderung [1]. Zusätzlich sind die oft stark eingeschränkte Prognose von onkologischen Patienten und deren immunologische Situation zu berücksichtigen, sodass Ausmaß und Invasivität der operativen Stabilisierung patientenindividuell angepasst werden müssen. Als chirurgische Optionen stehen die Azetabuloplastik [11], die sog. Harrington-Methode [6] und, bei entsprechend guter Überlebensprognose des Patienten, der Einsatz von Beckenteilersätzen zur Verfügung [2, 8]. Der Einsatz der minimal-invasiven Azetabuloplastik, dem Einbringen von PMMA (Polymethylmethacrylat) in Osteolyseherde des Azetabulumdachs, ist Situationen mit intakter vorderer und hinterer Säule sowie der medialen Wand vorbehalten (= Metastatic Acetabular Classification [MAC] 1, [3]) und verfügt daher über einen nur geringen Indikationsbereich. Das Einbringen von Steinmann-Nägeln in das Os ilium mit Verwendung von PMMA als Verbundosteosynthese (= Harrington-Methode [6]) und die anschließende Implantation einer Hüftpfanne ist eine seit Längerem bekannte Methode, die viele Variationen erfahren hat und auch bei Defekten der hinteren Säule angewendet werden kann (MAC 3, [7, 12, 14]). Allerdings ist bei der anterograden Einbringung der Steinmann-Nägel ein weiterer Zugang iliakal notwendig, und das Konstrukt birgt das Risiko einer Medialisierung aufgrund fehlender medialer Abstützung [5]. Die Implantation eines Beckenteilersatzes stellt eine hochinvasive Prozedur dar, die nur wenigen Patienten mit großen azetabulären Defekten und langer Überlebensprognose bzw. entsprechenden körperlichen Ressourcen vorbehalten ist. Gleichwohl bestehen ein hohes Infekt- und Komplikationsrisiko peri- und postoperativ [4, 8]. Dennoch sind unter Berücksichtigung der, durch neue Immun- und Hormontherapien, immer längeren Überlebenszeiten von Patienten mit Karzinomen Lösungen für eine Wiedererlangung von Mobilität, auch bei größeren Destruktionen des Hüftgelenkes anzubieten und zu entwickeln [10, 13]. In einer vorangegangenen Publikation derselben Arbeitsgruppe wurde die Anwendung der MRS-C-Abstützschale („MRS-TITAN® Comfort“, Peter Brehm GmbH, Weisendorf, Deutschland) in der Hüftrevisionsendoprothetik mit guten operativen und funktionellen Ergebnissen vorgestellt [15]. Das Implantat hat durch entsprechende prozedurale Modifikationen in der Folge eine Anwendungserweiterung in der metastatischen Situation des Azetabulums erfahren, welche nach Auffassung der Autoren Vorteile gegenüber den oben genannten Verfahren bietet. Während in der Revisionsendoprothetik die Abstützschale in Verbindung mit metallischen Augmenten eine lockerungs- und abriebbedingte Defektsituation des Azetabulums zementfrei überbrückt, steht in der metastatisch-lytischen Knochensituation der verbundosteosynthetische Charakter mit interdigitierender PMMA-Augmentation im Vordergrund. Im Folgenden wird daher die Möglichkeit der azetabulären, metastasenbedingten Defektrekonstruktion und Frakturstabilisierung mit einer zementaugmentierten Abstützschale (MRS‑C, Fa. Brehm) beschrieben.

## Operationsprinzip und Ziel

**Figure 1:**
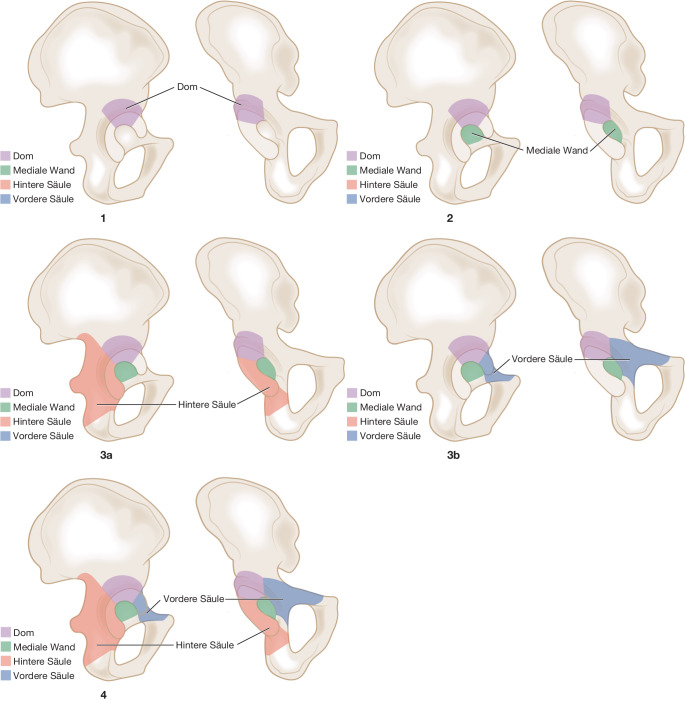

Mithilfe der MRS-C-Abstützschale können metastatische Defekte und pathologische Frakturen des vorderen und hinteren azetabulären Pfeilers sowie der medialen Wand und des Pfannendaches verbundosteosynthetisch über einen Standardhüftgelenkzugang überbrückt werden. Die Morphologie der Abstützschale mit einer zentralen Öffnung ermöglicht nach primärer Schraubenverankerung die Einbringung von PMMA in den Knochendefekt und eine vollständige Zementeinbettung der Schrauben. Voraussetzung ist eine zumindest noch teilweise vorhandene kraniale iliakale Kortikalis (Typ II und III nach Harrington [9], Typ 2–4 nach MAC; Tab. 1; Abb. 1). Anschließend erfolgt die Implantation der MRS-C-Abstützschale und einer in Inklination und Anteversion variablen, nicht zementierten Pfannenkomponente oder einer zementierten Polyethylen- bzw. tripolaren Pfanne. Durch die Rekonstruktion und Stabilisierung der azetabulären Anatomie ist eine zeitnahe Remobilisation des Patienten mit perspektivischer Vollbelastung und damit Wiedererlangung von Lebensqualität auch bei eingeschränkter onkologischer Prognose möglich.

**Table 1:** 

Typ I	Lateraler Kortikalisdefekt, superiore und mediale Wand intakt
Typ II	Medialer Wanddefekt
Typ III	Lateraler Erker- und Pfannendachdefekt
Typ IV	Isolierte Läsion mit kurativer Therapieintention und Resektion

## Vorteile

- Standardhüftgelenkzugänge ohne zusätzliche Zugangswege
- Gute Überbrückung von großvolumigen azetabulären Metastasen (MAC 2–4)
- Freie Positionierungsmöglichkeit der Pfannenkomponente in der Abstützschale durch Einzementierung einer Polyethylen- oder tripolaren Pfanne
- Im Vergleich zur Harrington-Methode weniger Gefahr der Konstruktmedialisierung durch kraniale polyaxiale Schraubenfixierung und kaudale Hakenaufhängung (bei noch vorhandenem Os pubis)
- Sofortige Vollbelastung und Remobilisation des Patienten
- Intraoperative „Customization“ durch multiple Möglichkeiten der Schrauben- und Pfannenpositionierung je nach Defektsituation
- Verwendung von modularen Standardinlays und/oder tripolaren Pfannensystemen nach Einbringung der Abstützschale

## Nachteile

- Hohes periimplantäres Infektrisiko bei Immunsuppression durch Chemotherapie und lokale Radiotherapie
- Keine Möglichkeit der Verankerung der Abstützschale bei großen kranialen iliakalen Defekten und dorsalen Pfeilerdefekten
- Anspruchsvolle Instrumentations- und Operationstechnik
- Knochennahe Dissektion des M. gluteus medius und minimus vom Os ilium zur Verankerung der Laschen notwendig

## Indikation

- Metastasenbedingte azetabuläre Defektsituationen vom Typ II und III nach Harrington [6, 9] und Typ 2–4 nach MAC [3] bei mittel- und langfristiger Überlebensprognose des Patienten (mindestens 3 Monate) und eingeschränkter Mobilisation

## Kontraindikationen

- Starke Einschränkung der Überlebensprognose (< 6 Wochen)
- Persistierendes lokales Infektgeschehen
- Ausgedehnte kraniale Defekte des Os ilium ohne Verankerungsmöglichkeit der Laschen- oder Erkerschrauben (s. Abschnitt „Implantat und Instrumentarium“)
- Ausgedehnte dorsale Pfeilerdefekte, ausgeprägte Beckendiskontinuität
- Grunderkrankungsbedingte Inoperabilität des Patienten
- Vorliegen eines primären Knochentumors mit kurativem Therapievorgehen
- Laufende wundheilungskompromittierende Chemo- oder Immuntherapie

## Patientenaufklärung

- Erhöhtes Blutungsrisiko durch Eröffnung der Metastase (cave: Nierenzellkarzinom)
- Allgemeine Risikoaufklärung über Beckeneingriffe
- Postoperativ hinkendes Gangbild (Trendelenburg-Hinken) durch Weichteilschädigung (Glutealmuskulatur)
- Beinlängenunterschied
- Protheseninstabilität/-luxation
- Implantatbruch/periprothetische Frakturen
- Periimplantäre Infektion (Früh- und Spätinfektion) insbesondere bei Immunsuppression
- Zementallergie
- Bei Übergewicht (BMI > 25 kg/m^2^; nach WHO-Definition) zusätzliche Aufklärung über „Off-label-Use“
- Verletzung von Gefäß‑/Nervenstrukturen mit postoperativer Durchblutungsstörung oder neurologischen Ausfällen
- Größenzunahme der Metastase und damit sekundäre Instabilität des Konstruktes
- Entgegen der vorher durchgeführten Schnittbildgebung können sich intraoperativ, insbesondere nach der Kürettage der azetabulären Metastase ausgedehnte Defekte oder Beckendiskontinuitäten zeigen, welche die Verwendung einer Abstützschale in der beschriebenen Technik unmöglich machen. Für diese Situation sollte mit dem Patienten präoperativ ein „Back-up-Vorgehen“ abgesprochen werden (z. B. Belassen einer Girdlestone-Situation, Einbringen eines Beckenteilersatzes). Das entsprechende Vorgehen sollte die Prognose und das Mobilisationspotenzial des Patienten berücksichtigen.

## Operationsvorbereitungen

- Konventionelle Röntgenaufnahmen mit Referenzierungsobjekt zur Prothesenplanung (Hüftübersicht a.-p. + Lauenstein-Aufnahme)
- Computertomographie lokal (Beurteilung des ventralen und dorsalen Pfeilers, Kontinuität des Beckens) und CT Thorax/Abdomen als „Staging“ zur Prognosebeurteilung des Patienten
- Interdisziplinärer Tumorboard-Beschluss
- Ggf. Pausierung von laufenden Immun‑/Chemotherapien soweit möglich (Wundheilung)
- Prüfung der Durchführung einer präoperativen, interventionellen Embolisation (Nierenzellkarzinom)
- Klinische Beurteilung der Weichteile und des Zugangsweges (Weichteilmetastasen?)

## Implantat und Instrumentarium

**Figure 2:**
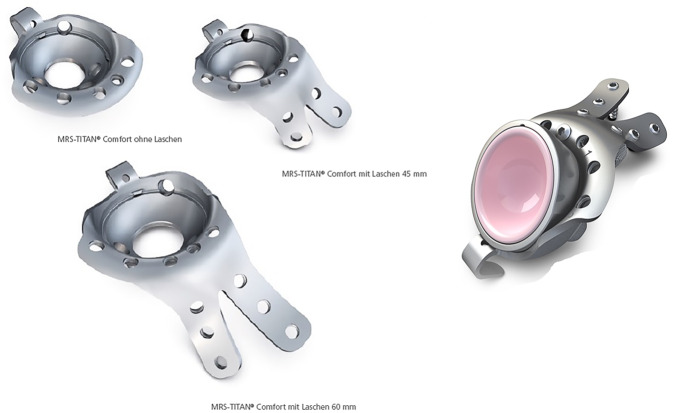

**Figure 3:**
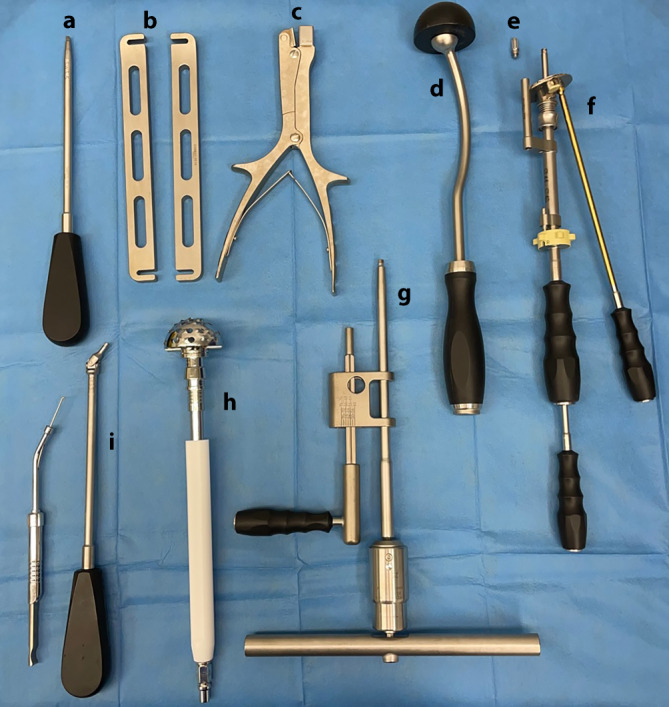

- Das Implantat (Abb. 2; mit freundlicher Genehmigung der Firma Brehm) besteht aus Reintitan mit einem hemisphärischen Außendurchmesser von 48–64 mm (in 4‑mm-Schritten) ohne und mit 2 verfügbaren kranialen Laschenlängen (45 und 60 mm). Aufgrund der oftmals vorliegenden kranialen metastatischen Osteolyse sind längere Laschen meist notwendig. Die sphärische Schale verfügt über eine Anteversion von 15° im Vergleich zur Doppellasche. Die Hemisphäre besitzt eine zentrale, verschließbare Öffnung, durch die Knochenzement im Sinne der Verbundosteosynthese eingebracht werden kann. Die zusätzlich implantierbare Pfannenkomponente ist in ihrer Inklination und Version in 6 Stufen einstellbar. Die Verankerungsschrauben sind polyaxial (Pfannendomschrauben) und winkelstabil (Laschenschrauben) einzubringen. Der kaudale Haken dient zur möglichst optimalen Rekonstruktion des Drehzentrums mit Positionierung im Foramen obturatorium. Er ist individuell modellierbar und kann optional entfernt werden. Das entsprechende Instrumentarium ist in Abb. 3 dargestellt.

## Anästhesie und Lagerung

- Rücken- oder Seitenlagerung, posterolateraler, transglutealer oder anterolateraler Zugang
- Blasendauerkatheteranlage
- Intubationsnarkose
- Tranexamsäure unter Beachtung der medikamentenspezifischen Kontraindikationen
- Steriles Abdecken des Röntgenbildwandlers (Inlet‑/Outlet-Aufnahmen)
- Single-Shot-Antibioseprophylaxe je nach Allergiesituation alle 3 h OP-Zeit

## Postoperative Behandlung

- Steriler Wundverband, tägliche klinische Wundkontrolle
- Postoperative Blutbildkontrolle mit Bestimmung von Hämoglobin (Hb) und C‑reaktivem Protein (CRP)
- Faden‑/Klammerentfernung nach 10 Tagen
- Sofortige Mobilisation unter schmerzadaptierter Vollbelastung, ggf. an Unterarmgehstützen
- Physiotherapie zur Kräftigung der hüftstabilisierenden Muskulatur
- Vermeidung von Außenrotation und Adduktion
- Adjuvante Radiatio nach Abschluss der primären Wundheilung
- Ggf. Fortsetzung der systemischen Therapie gemäß Tumorboard-Beschluss

## Operationstechnik

**Figure 4:**
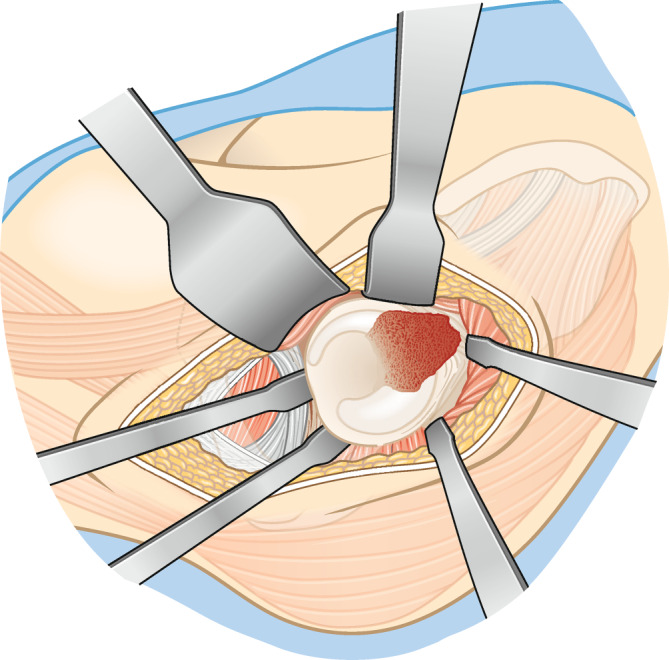

**Figure 5:**
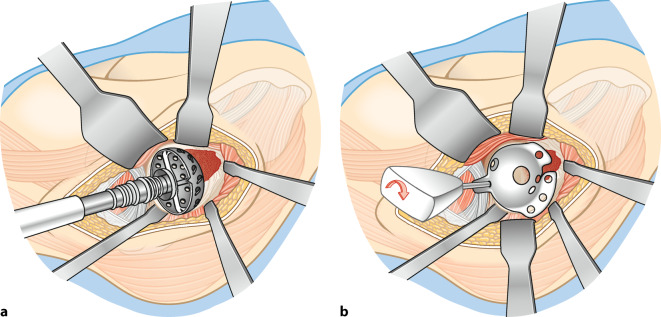

**Figure 6:**
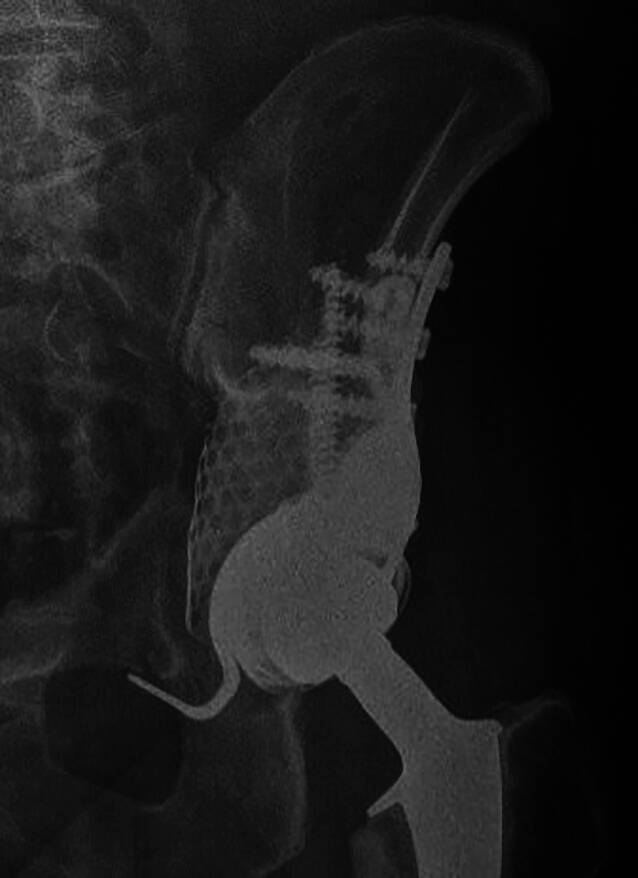

**Figure 7:**
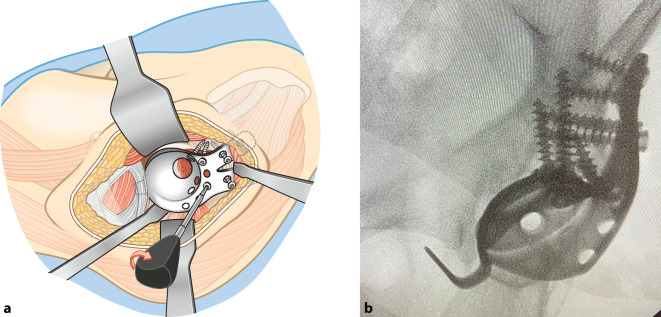

**Figure 8:**
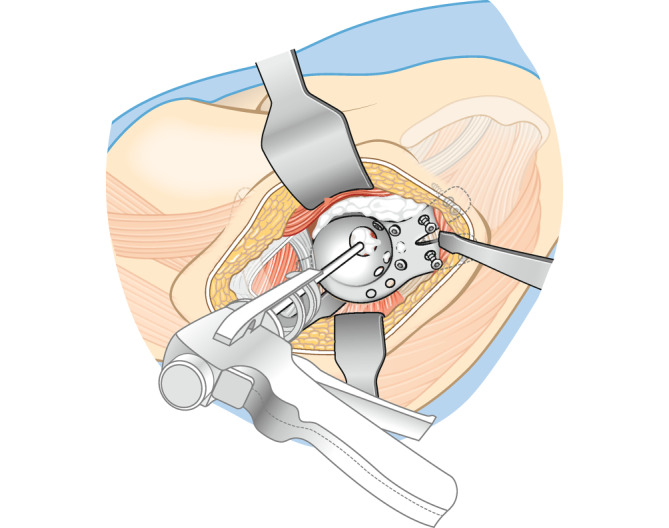

(Abb. 4, 5, 6, 7, 8, 9 und 10)

**Figure 9:**
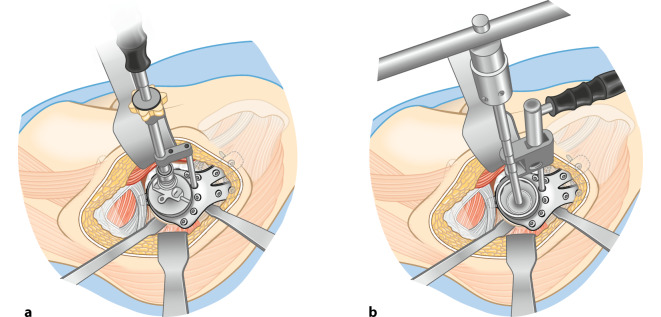

**Figure 10:**
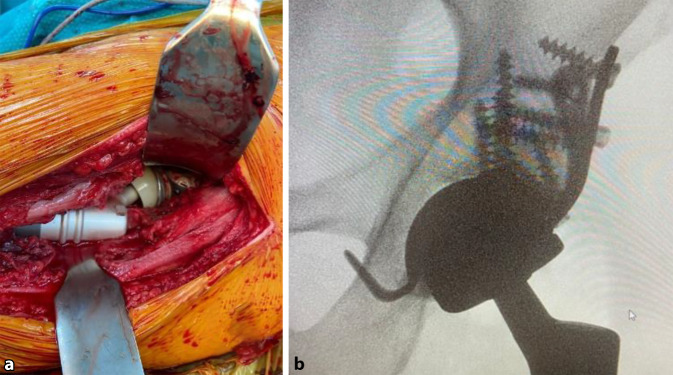

## Fehler, Gefahren und Komplikationen

- Verletzung der A. glutea superior bzw. deren abgehender Äste bei der Dissektion der Glutealmuskulatur vom Os ilium: Zugangserweiterung und Darstellung der Blutungsquelle, sodann Koagulation/Ligatur bzw. mikrochirurgische Gefäßnaht
- Resultierende Glutealinsuffizienz mit Trendelenburg-Hinken bei zugangsbedingtem Muskeltrauma: beckenstabilisierende physiotherapeutische Muskelkräftigung, individuelle orthopädische Hilfsmittelversorgung (z. B. gegenseitiger Gehstock)
- N.-femoralis-Läsion (Gefahr durch ventralen Haken am vorderen Azetabulumpfeiler) oder N.-ischiadicus-Läsion (Gefahr durch dorsalen Haken am hinteren Azetabulumpfeiler): neurologische Differenzialdiagnostik zur Schädigungsverifikation und -lokalisation (ggf. Elektromyographie und Nervenleitgeschwindigkeitsmessung), bei Restfunktion zunächst abwartendes Verhalten und physiotherapeutische Beübung zur Förderung der Reinnervation, neurologisch-neurochirurgische interdisziplinäre Beteiligung und Therapie in Abhängigkeit der Patientenprognose und Restfunktion
- Übermäßige Zementauffüllung und nicht vollständig versenkte Titan-Flachkopfspongiosaschrauben in der Abstützschale („Pfannendomschrauben“) behindern die Arretierung der Titaninnenpfanne und führen zu einer instabilen Verbindung des modularen Inlays in der Abstützschale (alternativ): Wechsel oder Nachziehen der Schrauben bzw. Entfernung des übermäßigen Zementes, alternativ Einzementierung einer PE-Pfanne in die Abstützschale
- Fehllage oder Überlänge der Pfannendom- und Laschenschrauben mit Affektion intrapelviner Gefäß‑, Nerven- und Organstrukturen: Kontrolle durch intraoperative BV-Aufnahmen in Inlet‑/Outlet-Technik und ggf. Wechsel der Schrauben

## Ergebnisse

**Figure 11:**
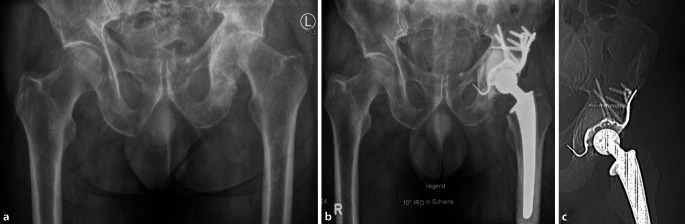

Im Rahmen einer retrospektiven Analyse erfolgte die Nachbeobachtung von 14 Patienten mit metastatischem Befall des Azetabulums, die zwischen 2012 und 2019 an unserer Klinik mittels einer PMMA-augmentierten MRS-C-Abstützschale stabilisiert wurden. Die Indikation zur operativen Intervention bestand in allen Fällen aufgrund einer vollständigen Immobilisierung durch eine pathologische Azetabulumfraktur oder einer therapieresistenten Schmerzsituation durch den azetabulären metastatischen Befall (Fallbeispiel: Abb. 11). Die zugrunde liegenden Tumorentitäten und epidemiologischen Daten wurden prä- und perioperativ erhoben. Das klinische Ergebnis wurde anhand der Mobilität und Beweglichkeit im Rahmen des Harris-Hip-Scores (HHS) und der dokumentierten postoperativen Komplikationen gemessen.

Das durchschnittliche Alter zum Operationszeitpunkt betrug 71,3 Jahre (52 bis 89 Jahre). Das Follow-up des Kollektivs betrug 1 bis 29 Monate (Mittel: 10,6 Monate). Die zugrunde liegenden Tumorentitäten waren Prostatakarzinom (*n* = 4), Mammakarzinom (*n* = 4) sowie Nierenzellkarzinom (*n* = 1), Urothelkarzinom (*n* = 1), multiples Myelom (*n* = 1), malignes Melanom (*n* = 1) und CUP („cancer of unknown primary“, 2). In allen Fällen lagen eine multiple Organ- und Lymphknotenmetastasierung vor. Die mittlere Operationsdauer betrug 209,1 ± 58,7 min. Zum Zeitpunkt der Datenerhebung waren 10 der 14 Patienten (71,4 %) verstorben. Das mittlere Überleben nach erfolgter Operation betrug 9,7 (1 bis 29) Monate, wobei die Standzeiten von 13 der 14 Implantate der Prognose des Patienten entsprachen und derjenigen aktuell noch unter Follow-up entsprechen. Das eingeschränkte Überlebensintervall der Patienten war auf den fortgeschrittenen Grad der Tumorerkrankung zurückzuführen. In einem Fall kam es bei kompromittierter Weichteilsituation zu einer periprothetischen Infektion und zum Ausbau der Prothese. In einem weiteren Fall musste aufgrund von rezidivierenden Luxationen das femorale Offset verlängert, die Hüftkomponente jedoch nicht verändert oder explantiert werden. Zum Zeitpunkt der Entlassung der Patienten verbesserte sich der Harris-Hip-Score im Mittel um 23,21 Punkte (0–38,21) im Vergleich zur präoperativen Situation.
